# Liquid–liquid phase separation of N-isopropylpropionamide aqueous solutions above the lower critical solution temperature

**DOI:** 10.1038/srep24657

**Published:** 2016-04-21

**Authors:** Kenji Mochizuki, Tomonari Sumi, Kenichiro Koga

**Affiliations:** 1 Department of Chemistry, Faculty of Science, Okayama University, Okayama 700-8530, Japan; 2 Research Institute for Interdisciplinary Science, Okayama University, Okayama 700-8530, Japan

## Abstract

We investigate driving forces of the liquid–liquid phase separation of N-isopropylpropionamide (NiPPA) aqueous solutions above the lower critical solution temperature using molecular dynamics simulations. Spontaneous phase separations of the model aqueous solution with a modified OPLS-AA force field are observed above the experimentally determined cloud point. The destabilization toward the phase separation is confirmed by temperature dependence of the long-wavelength limit of the concentration-concentration structure factor, the dominant component of which is found to be an increasing effective attraction between NiPPA molecules. At varying temperatures, the potentials of mean force (PMFs) between a pair of NiPPA molecules at infinite dilution are obtained and decomposed into the nonpolar and Coulombic contributions. The nonpolar contribution, arising essentially from molecular volume, promotes association of NiPPA molecules with increasing temperature while the Coulombic one antagonizes the association. Thus, our analysis leads to a conclusion that the driving force of thermally induced aggregation of NiPPA molecules is the temperature dependence of the nonpolar contribution in PMF between NiPPA molecules, not the temperature dependence of the number or strength of hydrogen bonds between NiPPA and water molecules.

Some classes of liquid mixtures exhibit a lower critical solution temperature (LCST), below which there is only one homogeneous phase and above which the homogeneous solution with a fixed composition undergoes phase separation into two coexisting liquid phases. The phase separation associated with LCST can be observed for aqueous solutions of small amphiphilic molecules^1^, such as lutidine^2^ and tetrahydrofuran^3^, but it is more commonly seen in aqueous polymers^4^,^5^. The heat-induced phase separation is an intriguing, counterintuitive phenomenon. Driving forces for phase separation at higher temperatures must be something that overcomes the opposite effect due to the entropy of mixing. Hirschfelder *et al.* explained the demixing of aqueous solutions above LCST in terms of the formation of hydrogen bonds between unlike molecules at lower temperatures and the destruction of them at higher temperatures^6^; the proposed mechanism has been widely used in constructing lattice models with LCST^7^,^8^,^9^. Continuum models with directional attractive interactions, based on the seminal work of Wertheim^10^,^11^, have been used to study closed-loop immiscibility^12^,^13^,^14^.

There is a different view about the exhibition of LCST. It has been noted that the temperature dependence of solubility of one species above LCST is a manifestation of the hydrophobic effect^15^. Extensive studies have shown that the water-mediated “indirect” interaction, or the hydrophobic interaction, must be taken into consideration to explain the association between hydrophobic solute molecules in water^15^,^16^, as well as the self-assembly of biomolecules and amphiphilic molecules^17^,^18^,^19^. A notable feature of the hydrophobic interaction between simple, nonpolar molecules in water is that the effective attractive interaction becomes stronger with increasing temperature^20^,^21^,^22^. However, the temperature dependence of the effective interaction, or the potential of mean force (PMF), between amphiphilic molecules in aqueous solutions has been little explored. One of unresolved issues is whether or not the existence of LCST in aqueous solutions may be explained in terms of the temperature dependence of the effective interactions between amphiphilic molecules.

The coil-to-globule transition of poly(N-isopropylacrylamide), a widely studied thermoresponsive aqueous polymer, has been explained by the dehydration of water molecules around isopropyl groups^23^,^24^,^25^. However, the relationship between the coil-to-globule transition and the phase separation is not clear; many factors affecting the phase behavior such as tacticity^26^,^27^ are not well understood; and a question of identifying the cloud point as the onset of macroscopic phase separation^28^ remains unresolved.

Computer simulations can provide molecular-level insight into phase transition behaviors, as demonstrated in the cases of water and aqueous solutions^29^,^30^,^31^,^32^; however, to date there are few simulation studies that demonstrate heat-induced spontaneous phase separation of aqueous amphiphilic solutions and try to answer the question as to which factors, hydrogen bonds between unlike molecules or the hydrophobic effect, play a critical role in demixing upon heating^33^,^34^,^35^,^36^.

N-isopropylpropionamide (NiPPA, Fig. 1) is one of the smallest molecules that exhibit LCST^37^,^38^ and its simple conformation is expected to provide fundamental information about this phenomenon. In addition, since NiPPA is the repeating unit of poly(N-isopropylacrylamide), investigation of NiPPA solutions will give important insights toward understanding the coil-to-globule transition and the phase separation behaviors of poly(N-isopropylacrylamide).

In this study, we demonstrate the spontaneous phase separation of aqueous solutions of NiPPA upon heating by performing molecular dynamics (MD) simulations with a modified OPLS-AA force field. Analyses of the long-wavelength limit of the concentration-concentration structure factor as obtained at varying concentrations quantitatively reveal that the solution is destabilized on approach to the phase-separation temperature and the destabilization is predominantly attributed to the decrease of the PMF curve between NiPPA molecules. We also discuss how the multi-body effect in the effective interaction between NiPPA molecules contributes to the phase stability. Then, we obtain the PMFs for a pair of NiPPA molecules in water at infinite dilution at temperatures over a wide range below and above the phase separation point, and decompose the PMF curves into nonpolar and Coulombic contributions in one case and into the direct and the water-mediated interactions in the other case. Then which factors are responsible for the tendency toward aggregation of NiPPA molecules with increasing temperature is discussed.

## Results and Discussions

### Spontaneous phase separation

**Figure 2:**
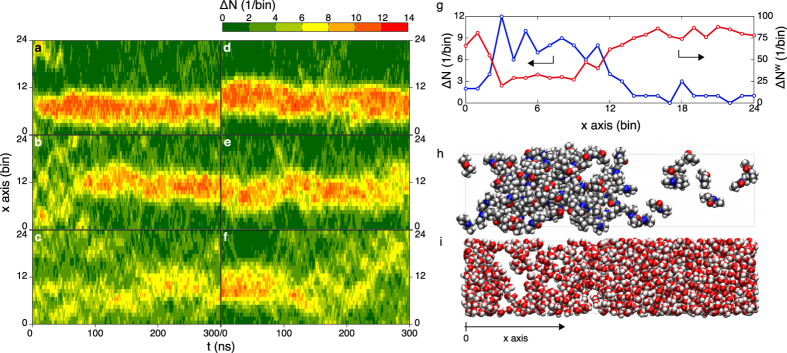
Local density fluctuations above and below the experimentally determined cloud point (~311 K). Time evolution of the local density of NiPPA molecules, Δ*N*(*x*, *t*), for the trajectories starting from a well-mixed configuration at (**a**) 340 K, (**b**) 320 K, and (**c**) 300 K and those starting from a phase-separated configuration at (**d**) 340 K, (**e**) 320 K, and (**f**) 300 K; (**g**) Local densities of NiPPA molecules, Δ*N*(*x*, *t*), and water molecules, Δ*N*^*W*^(*x*, *t*), in a state of the liquid–liquid phase separation at 340 K, as obtained at *t* = 200 ns in trajectory (a); Snapshots of (**h**) NiPPA molecules and (**i**) water molecules in the same state as (**g**).

To assess whether the macroscopic phase separation actually occurs in the model system of an aqueous solution of NiPPA, we performed long-time MD simulations in the isothermal-isobaric (*NPT*) ensembles of the 30 wt% solution in a rectangular cell with *L*_*x*_:*L*_*y*_:*L*_*z*_ = 4:1:1, which contains 100 NiPPA molecules and 1500 water molecules. The production run for each simulation is 300 ns. Use of the rectangular cell facilitates phase separation at temperatures above the cloud point. The local density of NiPPA molecules, Δ*N*(*x*, *t*), defined as the number of the centers of mass of NiPPA molecules found at time *t* in a slab of width Δ*x* centered at *x*, is computed from the MD trajectory, where Δ*x* ≡ *L*_*x*_/24 ≃ 4.2 Å^39^. Figure 2a–c show the time evolutions of Δ*N*(*x*, *t*) at *T* = 340, 320 and 300 K. The initial configurations at *t* = 0 are those of the homogeneous mixtures. At 300 K (Fig. 2c), numerous islands are present and fluctuations in the local density are observed. During a time range *t* = 150–270 ns, one can see a relatively-high-density domain in the background of low-density region, which appears to be indicative of liquid–liquid phase separation; after 270 ns, however, the contrast in high and low densities becomes less sharp. Thus it appears that no liquid–liquid phase separation occurs at 300 K. At 320 K (Fig. 2b), on the other hand, two distinct domains with high and low densities of NiPPA emerge at around *t* = 100 ns and persist afterward, indicating that spontaneous liquid–liquid phase separation actually occurs at this temperature. Movie S1 shows the aggregation dynamics of NiPPA molecules in 200 ns in the solution at 320 K (the MD trajectory is the same as used to draw Fig. 2b and water molecules are not shown in the movie). One can see after 85 ns NiPPA molecules start to aggregate around the center of the simulation box. At a higher temperature of 340 K (Fig. 2a), the initially homogeneous solution separates into two domains more promptly than at 320 K and the difference in NiPPA concentration between the two is larger than that at 320 K. Figure 2g shows spatial variations of Δ*N*(*x*, *t*) and the local density Δ*Ν*^*W*^(*x*, *t*) of water at 340 K in the system with two domains at a given instance (*t* = 200 ns), with Δ*Ν*^*W*^(*x*, *t*) defined in the same way as Δ*N*(*x*, *t*). Snapshots of the NiPPA and water molecules at that instance are also shown in Fig. 2h,i. These results clearly demonstrate the coexistence of two distinct phases in the simulation box: in Fig. 2g the liquid-liquid interfaces appear to be located at the third and 12th bins.

To check any dependence of the phase behavior on the initial configuration of the MD simulation, we conducted additional MD simulations starting from a phase-separated configuration at *t* = 0, as shown in Fig. 2d–f. At 300 K (Fig. 2f), the initial two-phase structure disappears after 100 ns and the NiPPA molecules are dispersed in the simulation box with large fluctuations in Δ*N*(*x*, *t*). Movie S2 presents the dynamics of NiPPA molecules at 300 K, in which one can see that aggregated molecules in the middle of the box are gradually dispersed and ultimately there appears a homogeneous solution (the MD trajectory is the same as shown in Fig. 2f). In contrast, at 320 K and 340 K, two domains continue to coexist until the end of the simulation at *t* = 300 ns. The results at respective temperatures indicate that after 100 ns homogeneous or inhomogeneous states of the solution that evolved from the unmixed state are the same as those which emerged from the mixed configuration in Fig. 2a–c, thereby suggesting that the MD simulations of 300 ns are sufficiently long to achieve equilibrium states.

In this study, we modified the OPLS-AA force field so that the macroscopic phase separation of the 30 wt% aqueous solution of model NiPPA occurs at the cloud point (~311 K) observed in experiment^37^. We increased all the original electrostatic charges on the interaction sites in NiPPA by a factor *χ* and examined phase behaviors of the 30 wt% aqueous solutions with *χ* = 1.40, 1.31, 1.30 and 1.00, the last of which corresponds to the original OPLS-AA force field. The solutions with *χ* = 1.30 and 1.00 exhibit macroscopic phase separations even at 300 K, while the solution with *χ* = 1.40 does not exhibit phase separation at 340 K. On the other hand, as demonstrated in Fig. 2 the model aqueous solution of NiPPA with *χ* = 1.31 is homogeneous below 300 K and becomes inhomogeneous above 320 K; this temperature range includes the experimentally observed cloud point. To determine the whole shape of a miscibility gap with a precise location for this model solution, further extensive simulations for other mass fractions will be required.

### Stability of mixtures

**Figure 3:**
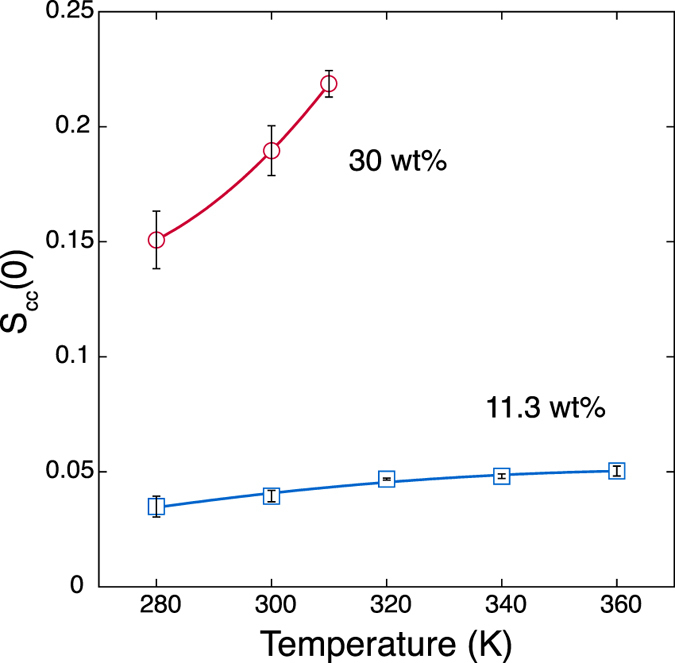
Temperature dependence of the long-wavelength limit of the concentration-concentration structure factor *S*_*cc*_(0) for the 11.3 wt% and 30 wt% solutions. The details of error bars are described in the method section.

**Figure 4:**
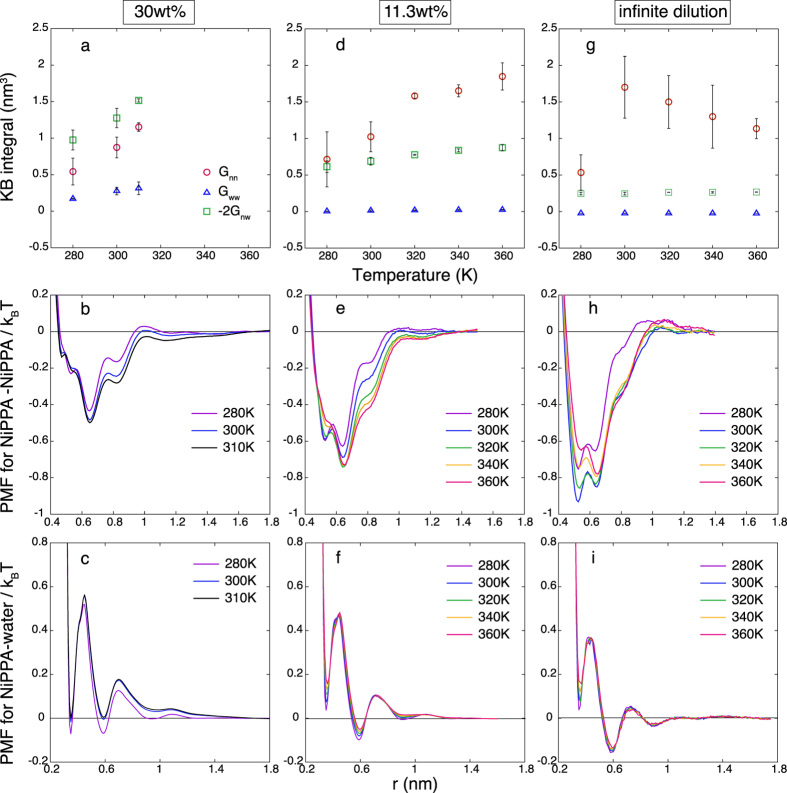
Concentration and temperature dependence of inter-molecular interactions: (**a**) the KB integrals (*G*_*nn*_, *G*_*ww*_ and −2*G*_*nw*_), (**b**) PMFs between NiPPA molecules, and (**c**) PMFs between NiPPA and water molecules for the 30 wt% solution; (**d**–**f**) those for the 11.3 wt% solution; (**g**–**i**) for the infinitely dilute solution.

The mixing stability of a binary mixture is described by the long-wavelength limit of the concentration-concentration structure factor *S*_*cc*_(0) ≡ lim_*q*→0_*S*_*cc*_(*q*)^40^,^41^,^42^,^43^,^44^, because *S*_*cc*_(0) diverges as the thermodynamic state approaches a stability limit for a homogeneous mixture. *S*_*cc*_(0) represents the mean square fluctuation in the particle concentration and is expressed by a linear combination of the Kirkwood-Buff (KB) integrals *G*_*ij*_^41^:


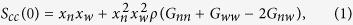


where *x*_*i*_ is the number concentration of the *i*th species and *ρ* is the average number density. Subscripts *n* and *w* represent NiPPA and water, respectively. The KB integrals for the 30 wt% aqueous solution of NiPPA are obtained from the *NPT*-MD simulations at 280, 300 and 310 K, at which the phase separation does not occur (Fig. 2). In Fig. 3, the increase of *S*_*cc*_(0) indicates that the mixture is unstabilized as temperature increases from 280 to 310 K. This result is consistent with what we observed in Fig. 2, where the phase separation temperature is estimated between 300 and 320 K. Because *x*_*n*_ and *x*_*w*_ are constant and *ρ* in Eq. (1) decreases from 280 to 310 K, the *G*_*ij*_s are responsible for the increase of *S*_*cc*_(0). Figure 4a shows that *G*_*nn*_ and −2*G*_*nw*_ predominantly contribute to the increase of *S*_*cc*_(0). In contrast, the contribution from *G*_*ww*_ is small. Furthermore, the increases of *G*_*nn*_ and −2*G*_*nw*_ are attributed to the decrease in the PMF curve between NiPPA molecules and the increase in the PMF curve between NiPPA and water molecules, respectively (Fig. 4b,c).

### Concentration dependence

The phase behaviors for the 11.3 wt% solution and the infinitely dilute solution are investigated in the same manner as the 30 wt% solution. The KB integrals for the 11.3 wt% solution are obtained from the normal *NPT*-MD simulations, and those for the infinitely dilute solution are obtained from the umbrella sampling simulations with the free weighted histogram analysis method^45^. Although the macroscopic phase separation does not occur at 11.3 wt%^37^, the increase of *S*_*cc*_(0) in Fig. 3 indicate that the 11.3 wt% solution is destabilized with increasing temperature, as seen for the 30 wt% solution. However, the temperature dependence of *S*_*cc*_(0) for the 11.3 wt% solution is much weaker than that for the 30 wt% solution. Figure 4d shows that *G*_*nn*_ is predominantly responsible for the destabilization of the 11.3 wt% solution, while the contribution from −2*G*_*nw*_ is secondary and that from *G*_*ww*_ is negligibly small. Furthermore, the changes of *G*_*nn*_ and −2*G*_*nw*_ are attributed to the change of the corresponding PMF curves. The minimum of PMF curve between NiPPA molecules becomes deeper as temperature increases from 280 to 360 K, while the PMF curve between NiPPA and water molecules becomes shallower, as shown in Fig. 4e,f. At infinite dilution, the stability of solution can not be evaluated, because *S*_*cc*_(0) = 0 from Eq. (1) with *x*_*n*_ = 0. Figure 4g shows *G*_*nn*_ at infinite dilution increases with increasing temperature from 280 to 300 K, as seen for the 11.3 and 30 wt% solutions. The PMF curves are similar to those at 11.3 wt% (Fig. 4h): both have a large basin with two local minima. However, there are some notable differences. *G*_*nn*_ at infinite dilution gradually decreases in the temperature range from 300 to 360 K, unlike at 11.3 wt%. The PMF between NiPPA molecules also show a non-monotonic change: the minimum becomes deeper as temperature increases from 280 to 300 K, and reversely the minimum becomes shallower as temperature increases further. The temperature range where the PMF curve becomes deeper with increasing temperature is larger for the 11.3 wt% solution (280–360 K) than for the infinitely dilute solution (280–300 K). This implies that the multi-body effect at finite concentrations^46^,^47^,^48^ plays some role in the phase separation.

Another remarkable feature is that the contribution to the phase stability from −2*G*_*nw*_ diminishes as the NiPPA concentration decreases. At infinite dilution, both −2*G*_*nw*_ and the PMF between NiPPA and water molecules vary very little with increasing temperature (Fig. 4g,i). On the other hand, *G*_*nn*_ and the PMF between NiPPA molecules exhibit significant temperature dependences at any concentration. These results imply that the temperature-induced destabilization of the NiPPA solution essentially results from the temperature dependence of the PMF between NiPPA molecules, rather than the PMF between NiPPA and water molecules. Then, the aggregation of NiPPA molecules may subsequently lead to the separation between NiPPA and water molecules at finite dilution.

### Decomposition of the potential of mean force

**Figure 5:**
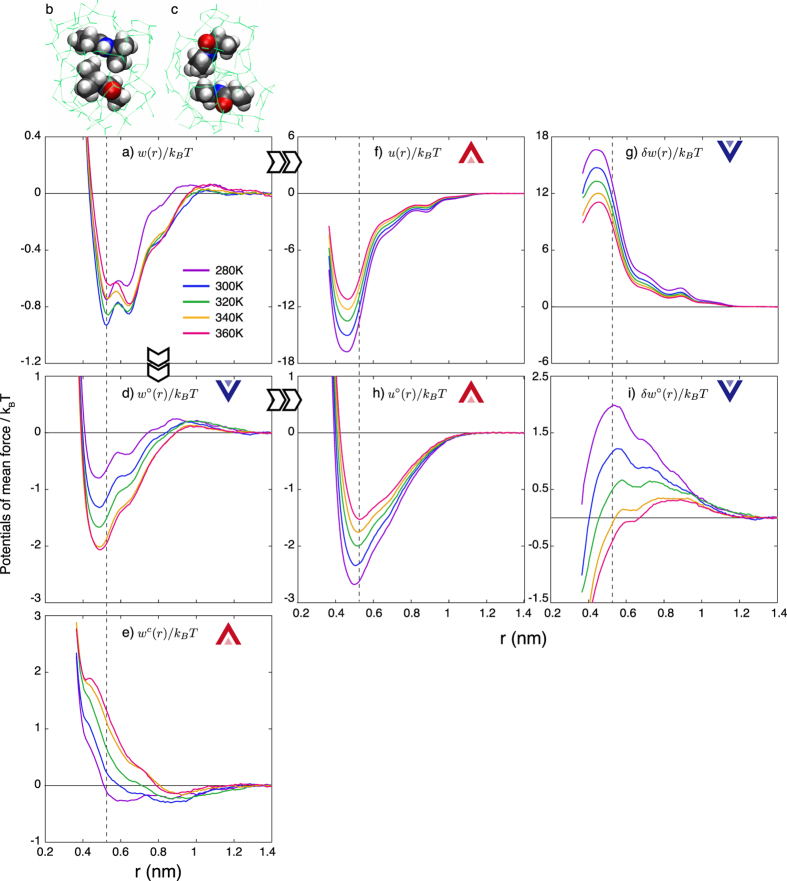
PMFs between NiPPA molecules at temperatures between 280 K and 360 K: (**a**) PMFs for NiPPA molecules in water, *w*(*r*)/*k*_*B*_*T*, and the typical configurations of a pair of NiPPA molecules (**b**) at the contact (*r*_*c*_ ≡ 0.53 nm) and (**c**) at the partial-contact distances (*r*_*pc*_ ≡ 0.64 nm), as obtained from the samplings at 280 K (Water molecules within 5 Å from any atom of NiPPA molecules and their hydrogen bond networks are represented by light green lines); the decomposition of *w*(*r*)/*k*_*B*_*T* into (**d**) the nonpolar contribution, *w*°(*r*)/*k*_*B*_*T*, and (**e**) th**e** Coulombic contribution, *w*^*c*^(*r*)/*k*_*B*_*T*; the decomposition of *w*(*r*)/*k*_*B*_*T* into (**f**) the direct interaction, *u*(*r*)/*k*_*B*_*T*, and (**g**) the water-mediated interaction, *δw*(*r*)/*k*_*B*_*T*; and the decomposition of *w*°(*r*)/*k*_*B*_*T* into (**h**) *u*°(*r*)/*k*_*B*_*T* and (**i**) *δw*°(*r*)/*k*_*B*_*T*. The same color is used for the same temperature in panels (**a**,**d**–**i**). Blue or red arrows represent the association or the dissociation behavior of the decomposed factors and the vertical dashed lines indicate the contact distance *r*_*c*_.

We now consider which factor is primarily responsible for the decrease of the local minima in PMFs between NiPPA molecules with increasing temperature. Figure 5a shows the PMF curves *w*(*r*)/*k*_*B*_*T* between a pair of NiPPA molecules in water at infinite dilution, which is the enlarged figure of Fig. 4h. The *w*(*r*)/*k*_*B*_*T* have a large basin with two local minima at *r*_*c*_ and *r*_*pc*_, corresponding to “contact” and “partial-contact” distances for the pair. At the lowest temperature, *r*_*c*_ = 0.53 nm and *r*_*pc*_ = 0.64 nm and the locations change only slightly with increasing temperature. Figure 5b,c illustrate typical configurations of a pair of NiPPA molecules at these distances, as obtained from the umbrella samplings at 280 K.

We divide the total PMF *w*(*r*) into the PMF *w*°(*r*) between the nonpolar NiPPA molecules in water and the Coulombic contribution *w*^*c*^(*r*), which is defined as *w*^*c*^(*r*) ≡ *w*(*r*) − *w*°(*r*)^49^. Figure 5d shows that *w*°(*r*)/*k*_*B*_*T* around the basin monotonically decreases with increasing temperature. This indicates that a factor simply stemming from the molecular volume of NiPPA contributes to stabilizing the association of NiPPA molecules in water at higher temperatures. Such temperature dependence, i.e., water-mediated attractive interaction being stronger with increasing temperature, has also been reported for simple nonpolar solutes^20^,^21^. In contrast, as shown in Fig. 5e, *w*^*c*^(*r*)/*k*_*B*_*T* monotonically increases with increasing temperature, promoting the dissociation of NiPPA molecules at higher temperatures. Both *w*°(*r*) and *w*^*c*^(*r*), when measured at *r*_*c*_, change approximately by 1.5 *k*_*B*_*T* and do so in the opposite direction when temperature is varied from 280 K to 360 K, resulting in that the total PMF *w*(*r*) varies less than 0.3 *k*_*B*_*T*.

The Coulombic contribution *w*^*c*^(*r*), i.e., the difference *w*(*r*) − *w*°(*r*), may be considered as the combined effects of the formation of hydrogen bonds between NiPPA and water and between NiPPA molecules and the reorientation free energy of water molecules. It has been suggested that the phase separation at higher temperatures for solutions with LCST is explained by entropy effects destroying the directional bonds, i.e., hydrogen bonds, between unlike molecules^6^,^7^,^8^,^9^. If this explanation were indeed true for the NiPPA solution, *w*^*c*^(*r*) would promote association of NiPPA molecules at higher temperatures. However, Fig. 5e shows the opposite effect, i.e., the Coulombic contribution to *w*(*r*) antagonizes the association more strongly at higher temperatures; it is *w*°(*r*) that promotes the association.

We next consider another kind of decomposition of *w*(*r*), which divides the total PMF *w*(*r*) into the PMF *u*(*r*) in vacuum (the direct interaction) and the water-mediated (indirect) interaction *δw*(*r*): the former is obtained by MD simulation of a pair of NiPPA in vacuum and the latter by subtraction *δw*(*r*) ≡ *w*(*r*) − *u*(*r*). Plotted in Fig. 5f are *u*(*r*)/*k*_*B*_*T* curves at varying temperatures, which have a local minimum between *r* = 0.4 and 0.5 nm, which is very large and negative. The *u*(*r*) is a sum of the dispersion and electrostatic interactions between NiPPA molecules, but the Coulombic interactions are dominant as can be seen from comparison of Fig. 5f with Fig. 5h. On the other hand, *δw*(*r*)/*k*_*B*_*T* has a large positive value, as shown in Fig. 5g, indicating that water as a solvent obstructs the association of NiPPA molecules. This effect is in accord with the earlier observation that the effective attraction between methane molecules in water is weaker than in vacuum at low and moderate temperatures^22^. The water-mediated interaction is given by 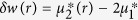, where 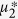 is the excess chemical potential of a pair of solute molecules with the intermolecular distance fixed at *r* and 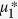 is that of an isolated solute molecule^50^. Thus, the large positive value of *δw*(*r*) as shown in Fig. 5g means that the solubility of the pair of NiPPA molecules is much lower than twice the solubility of an isolated NiPPA molecule.

The temperature dependences of direct and water-mediated interaction terms, *u*(*r*)/*k*_*B*_*T* and *δw*(*r*)/*k*_*B*_*T*, are much larger than the full PMF *w*(*r*)/*k*_*B*_*T*, that is, they are in the opposite directions and largely canceling each other: each varies by ~4.5 while the sum only by ~0.3 as temperature is increased from 280 to 360 K; and *u*(*r*)/*k*_*B*_*T* increases and *δw*(*r*)/*k*_*B*_*T* decreases with increasing temperature, as indicated by arrows in Fig. 5f,g. This means that the temperature dependence of the water-mediated interaction is responsible for the aggregation upon heating.

We now decompose the PMF *w*°(*r*) between the hypothetical nonpolar NiPPA molecules in water into the direct *u*°(*r*) and the water-mediated *δw*°(*r*) interaction terms. Figure 5h,i show that the direct part *u*°(*r*)/*k*_*B*_*T* becomes less negative but the water-mediated part *w*°(*r*)/*k*_*B*_*T* becomes lower with increasing temperature, as do *u*(*r*)/*k*_*B*_*T* and *δw*(*r*)/*k*_*B*_*T*. It is noted that the temperature dependences of *u*°(*r*)/*k*_*B*_*T* and *δw*°(*r*)/*k*_*B*_*T* for nonpolar NiPPA are weaker than those of *u*(*r*)/*k*_*B*_*T* and *δw*(*r*)/*k*_*B*_*T* for NiPPA. For example, at *r*_*c*_ both *u*(*r*)/*k*_*B*_*T* and *δw*(*r*)/*k*_*B*_*T* change by approximately 4.5 as temperature is varied from 280 K to 360 K, while *u*°(*r*)/*k*_*B*_*T* changes by 3.3 and *δw*°(*r*)/*k*_*B*_*T* by 2.4 with the same temperature variation. However, the total PMF *w*(*r*) for NiPPA exhibits a weaker temperature dependence than *w*°(*r*) for nonpolar NiPPA.

Figure 5i shows that *δw*°(*r*_*c*_)/*k*_*B*_*T* is positive at lower temperatures; but it monotonically decreases with increasing temperature and finally becomes negative at 340 K. The water-mediated part *δw*°(*r*_*c*_) between the nonpolar NiPPA molecules in water being repulsive at lower temperatures is different from what is known for methane in water; however this behavior is explained by the size-scale dependence of the water-mediated interaction^51^. The size-scale dependence is also supported by the fact that *δw*°(*r*_*c*_) = 2.9 kJ/mol at 300 K, as estimated in this study, is similar to the corresponding water-mediated interaction at the contact minima at 298 K for a pair of bicyclooctane molecules in water, 3.5 kJ/mol^51^, where the bicyclooctane molecule consists of eight carbon atoms and the molecular weight of 110 g/mol is similar to that of NiPPA (115 g/mol).

## Summary

Spontaneous liquid–liquid phase separation of NiPPA-water mixtures at higher temperatures was demonstrated by MD simulations of the model 30 wt% solution with a modified OPLS-AA force field. Temperature dependence of *S*_*cc*_(0), the long-wavelength limit of the concentration-concentration structure factor, confirms that the aqueous solution is destabilized as approaching the phase separation temperature. At 30 wt%, the aggregation between NiPPA molecules and the separation between NiPPA and water molecules, evaluated from the KB integrals, contribute almost equally to the destabilization. However, as the NiPPA concentration decreases, the contribution from the aggregation between NiPPA molecules becomes dominant. The comparison between 11.3 wt% and infinitely dilute solutions reveals that the multi-body effect enlarges the temperature range where the PMF curve between NiPPA molecules becomes deeper with increasing temperature.

To understand the driving force of the decrease of PMF curve between NiPPA molecules, the PMF *w*(*r*) at infinite dilution is decomposed into the nonpolar and Coulombic contributions. It is found that the nonpolar contribution *w*°/*k*_*B*_*T* becomes more negative, i.e., strengthens attraction, upon heating, while the Coulombic contribution *w*^*c*^/*k*_*B*_*T* becomes more positive or lessens attraction. The temperature dependence of the factor arising essentially from the molecular volume of solute molecules (the nonpolar contribution) is consistent with the earlier studies for simple nonpolar solutes^20^,^21^. The tendency of aggregation of nonpolar molecules, large or small, is generally enhanced with increasing temperature. On the other hand, the Coulombic factor is necessary to dissolve NiPPA in water at lower temperatures and to moderate the temperature dependence of PMF. Our analysis leads to a conclusion that the driving force of thermally induced aggregation of NiPPA molecules is the temperature dependence of the nonpolar contribution in PMF, which stems from the molecular volume of solute molecules, not the temperature dependence of the number or strength of hydrogen bonds between NiPPA and water molecules.

Alternatively, the PMF *w*(*r*) may be decomposed into the direct and the water-mediated interactions, *u*(*r*) and *δw*(*r*). The direct interaction energy divided by *k*_*B*_*T* monotonically increases with increasing temperature, i.e., becomes less attractive; the water-mediated contribution divided by *k*_*B*_*T*, which is positive, decreases upon heating. The temperature dependences of the direct and water-mediated interaction energy divided by *k*_*B*_*T* are much larger than that of the sum (*w*(*r*)/*k*_*B*_*T*), that is, the two contributions largely cancel each other, but the water-mediated contribution slightly prevails over the direct one.

With the model NiPPA solution, it has been shown that the temperature dependence of *G*_*nn*_ at infinite dilution (up to around 300 K) is qualitatively the same as those of *G*_*nn*_ at finite concentrations. The latter continue to increase at higher temperatures due to the multi-body effect and become the dominant factor in determining the temperature dependence of *S*_*cc*_(0) in Eq. (1) which should diverge at the stability limit of a homogenous phase. Further investigation is needed to determine quantitative conditions for the temperature dependent PMF between NiPPA molecules at finite concentrations that results in LCST near the room temperature. The temperature dependence of the PMFs for NiPPA as the repeating unit of poly(N-isopropylacrylamide) could well be an important factor that helps to understand the thermally-induced coil-to-globule transition of poly(N-isopropylacrylamide) in water^49^.

## Methods

### Force fields

The intermolecular interaction of water is of the TIP4P/2005 model^52^, which provides the most realistic description of the bulk liquid density and the excess chemical potential of simple molecules^53^. The model of NiPPA is based on the all-atom optimized parameters for liquid simulations (OPLS-AA) force field^54^, but with all the original electrostatic charges on the interaction sites in NiPPA increased by a factor of *χ* = 1.31^49^. We also performed *NPT*-MD simulations with *χ* = 1.40, 1.30 and 1.00 (the original OPLS-AA force field), then we chosen *χ* = 1.31 as the most appropriate one to reproduce the phase transition temperature of the 30 wt% NiPPA solution, as described in *Results and Discussions*. The parameters for non-bonded interactions for NiPPA are shown in Table 1. The Lennard–Jones parameters of the cross-interaction are computed by 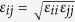 and 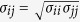. The intermolecular interactions are truncated at 9.0 Å . The long-range Coulombic interactions are evaluated by the particle-mesh Ewald algorithm^55^, and dispersion corrections are implemented for the energy and pressure evaluations.

### MD simulations

MD simulations in the canonical (*NVT*) and the isothermal–isobaric (*NPT*) ensembles are performed using the GROMACS 4.6.6. suite^56^. The time step of the MD simulations is 1.0 fs. The pressure of 0.1 MPa and the temperatures ranging from 280 K to 360 K are controlled by the Nośe–Hoover thermostat^57^,^58^ and the Parrinello–Rahman barostat^59^, respectively, whereas the Berendsen algorithm^60^ is used for equilibration. Periodic boundary conditions are applied for all directions of the simulation box. The lengths of equilibration and production runs depend on the system and the kind of analysis; details are given below and in *Results and Discussions*.

### PMFs and KB integrals at 11.3 wt% and 30 wt%

*NPT*-MD simulations of 30 wt% solution are performed at 280, 300 and 310 K. The solution consists of 100 NiPPA molecules and 1500 water molecules in a cubic box. The production run at each condition is 150 ns. The radial distribution functions *g*_*ij*_(*r*) between the centers of mass of NiPPA molecules, between the center of mass of NiPPA molecule and oxygen atom in water, and between oxygen atoms in water are first computed. For a closed system *g*_*ij*_(*r*) converges to a value less than 1 as *r* → ∞, so *g*_*ij*_(*r*) is corrected so as to become asymptotically 1 on average over the range *r* = 1.6 to 1.8 nm^22^. The same correction is also made for the radial distribution functions used for the KB integrals below. Then, the PMFs in Fig. 4b,c are obtained from the identity 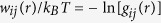. To obtained the KB integral *G*_*ij*_, the distance *R* dependence of the KB integral *G*_*ij*_(*R*) is first computed from the radial distribution function *g*_*ij*_(*r*), i.e., 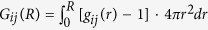. Then, *G*_*ij*_ is estimated as an average of *G*_*ij*_(*R*) over *R* = 1.6 to 1.8 nm. The range of *r* where *g*_*ij*_(*r*) is corrected to be 1 or the corresponding PMF is taken to be 0 is chosen to be located at a farthest distance in a given system. Although the choice is somehow arbitrary and depends on the system size, we confirmed that the temperature dependence of PMFs and *G*_*ij*_s obtained with a different region of 1.4–1.6 nm showed qualitatively the same results.

*NPT*-MD simulations of 11.3 wt% solution are performed at temperatures 280 K to 360 K with intervals of 20 K. The solution consists of 20 NiPPA molecules and 1000 water molecules in a cubic box. The PMFs and *G*_*ij*_s in Fig. 4d–f are computed in the same manner described above, where the base line, i.e., the PMFs being 0, is chosen at the region from 1.3 to 1.5 nm.

The error bars of each *G*_*ij*_ in Fig. 4a,d,g are the standard deviation of the block averages with the total run of 150 ns divided into three blocks. Then, the error bar of each *S*_*cc*_ in Fig. 3 is calculated as the propagation of error from the standard deviations of *G*_*nn*_, *G*_*ww*_ and *G*_*nw*_.

### PMFs and KB integrals at infinite dilution

PMFs in infinitely-dilute solutions are obtained from a set of umbrella sampling simulations with the free weighted histogram analysis method^45^, as described in our previous paper^49^. The system contains two NiPPA molecules and 1500 water molecules in a cubic box. PMFs between the center of mass of a NiPPA molecule and an oxygen atom of water and between oxygen atoms of water in Fig. 4h,i are computed through the corresponding radial distribution functions, which are obtained from the trajectory of the umbrella sampling simulation with *d*_0_ = 1.44 nm.

The *R* dependence of the KB integral *G*_*ij*_ is computed from the corresponding PMF *w*_*ij*_(*r*) via 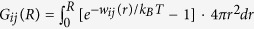. Then, *G*_*ij*_ is estimated as an average of *G*_*ij*_(*R*) over *R* = 1.3 to 1.4 nm. The error bar of each *G*_*nn*_ in Fig. 4g is computed as follows: (1) the umbrella sampling simulation of 30 ns at each *d*_0_ is divided into three block of 10 ns, (2) three PMF curves are computed from the three sets of the umbrella sampling simulations with the free weighted histogram analysis method^45^, (3) three *G*_*nn*_s and their standard deviation are computed. The error bar of each *G*_*ww*_ and *G*_*nw*_ in Fig. 4g is computed in the same manner as that for 11.3 and 30 wt% solutions.

In order to understand the temperature dependence of PMFs also computed are PMFs *u*(*r*) between NiPPA molecules in vacuum, PMFs *w*°(*r*) between hypothetical “nonpolar” NiPPA molecules without partial charges on the atoms in water, and PMFs *u*°(*r*) between those hypothetical NiPPA in vacuum. The method is similar to that for *w*(*r*) described above. The differences are in the cases of calculating *u*(*r*) and *u*°(*r*), where we perform *NVT* stochastic dynamics simulations with a production run of 10 ns for each condition and the cut-off scheme of 0.9 nm for the Coulombic interactions. The Coulombic contribution, *w*^*c*^(*r*), is defined by the difference between *w*(*r*) the PMF between NiPPA and *w*°(*r*) the PMF between the nonpolar NiPPA, i.e., *w*^*c*^(*r*) ≡ *w*(*r*) − *w*°(*r*). The water-mediated interactions between NiPPA molecules and between nonpolar NiPPA molecules are given by *δw*(*r*) ≡ *w*(*r*) − *u*(*r*) and *δw*°(*r*) ≡ *w*°(*r*) − *u*°(*r*), respectively.

## Additional Information

**How to cite this article**: Mochizuki, K. *et al.* Liquid–liquid phase separation of N-isopropylpropionamide aqueous solutions above the lower critical solution temperature. *Sci. Rep.* **6**, 24657; doi: 10.1038/srep24657 (2016).

## Supplementary Material

Supplementary Video 1

Supplementary Video 2

Supplementary Information

## Figures and Tables

**Figure 1:**
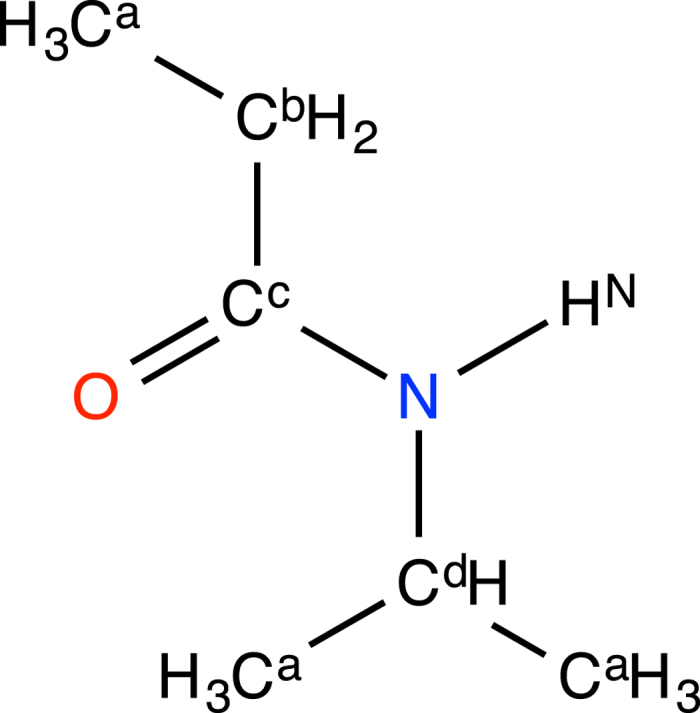
Chemical structure of NiPPA. The superscripts of carbon and hydrogen atoms represent the identification of the non-bonded parameters in Table 1.

**Table 1:** Lennard–Jones parameters *σ* and *ε* and electrostatic charges *q* for the interaction sites (atoms) in a NiPPA molecule.

Label	*σ* [Å]	*ε* [J/mol]	*q* [e]
H	2.50	125.520	0.0786
H^N^	0.00	0.000	0.3930
N	3.25	711.280	−0.6550
O	2.96	878.640	−0.6550
C^a^	3.50	276.144	−0.2358
C^b^	3.50	276.144	−0.1572
C^c^	3.75	439.320	0.6550
C^d^	3.50	276.144	0.1834

The labels are the same as those in Fig. 1.
