# Supplementary material for: Liquid–liquid phase separation of N-isopropylpropionamide aqueous solutions above the lower critical solution temperature
Source: Sci Rep. 2016 Apr 21;6:24657. doi: 10.1038/srep24657 (PMC4838917; doi:10.1038/srep24657)
Supplement: Supplementary Information [file srep24657-s3.pdf]

Supplemental Information for

Liquid-liquid phase separation of N-isopropylpropionamide aqueous solutions above the lower critical solution temperature

K. Mochizuki, T. Sumi and K. Koga

Department of Chemistry, Faculty of Science, Okayama University,
Okayama 700-8530, Japan

Supplemental Movie S1

This movie shows the aggregation dynamics of NiPPA molecules in the aqueous 30 wt% solution at 320 K. The MD trajectory is the same as used to draw Fig. 2b. Water molecules are not shown in the movie. The initial configuration is a homogeneous mixture. After 85 ns (19s in the video), one can see that NiPPA molecules start to aggregate around the center of the simulation box.

Supplemental Movie S2

This movie shows the dispersion dynamics of NiPPA molecules in the aqueous 30 wt% solution at 300 K. The MD trajectory is the same as used to draw Fig. 2f. Water molecules are not shown in the movie. The initial configuration is a phase separated one. One can see the aggregated molecules in the middle of the box are gradually dispersed and ultimately there appears a homogeneous solution.
